# Evaluation of the interaction between tumor growth factor-β and interferon type I pathways in patients with COVID-19: focusing on ages 1 to 90 years

**DOI:** 10.1186/s12879-023-08225-9

**Published:** 2023-04-18

**Authors:** Mitra Abbasifard, Ali Hasani Fakhrabadi, Fatemeh Bahremand, Hossein Khorramdelazad

**Affiliations:** 1 Immunology of Infectious Diseases Research Center, Research Institute of Basic Medical Sciences, Rafsanjan University of Medical Sciences, Rafsanjan, Iran; 2 Department of Internal Medicine, Ali-Ibn-Abi-Talib Hospital, Rafsanjan University of Medical Sciences, Rafsanjan, Iran; 3 Department of Immunology, School of Medicine, Iran University of Medical Sciences, Tehran, Iran

**Keywords:** COVID-19, TGF-β, Interferon, Fibrosis, SERPINE1

## Abstract

**Background:**

Evidence revealed that age could affect immune responses in patients with the acute respiratory syndrome of coronavirus 2 (SARS-CoV-2) infection. This study investigated the impact of age on immune responses, especially on the interaction between the tumor growth factor-β (TGF-β) and interferon type-I (IFN-I) axes in the pathogenesis of novel coronavirus disease 2019 (COVID-19).

**Methods:**

This age-matched case–control investigation enrolled 41 COVID-19 patients and 40 healthy controls categorized into four groups, including group 1 (up to 20 years), group 2 (20–40 years), group 3 (40–60 years), and group 4 (over 60 years). Blood samples were collected at the time of admission. The expression of *TGF-βRI*, *TGF-βRII*, *IFNARI*, *IFNARII*, interferon regulatory factor 9 (*IRF9*), and SMAD family member 3 (*SMAD3*) was measured using the real-time PCR technique. In addition, serum levels of TGF-β, IFN-α, and SERPINE1 were measured by the enzyme-linked immunosorbent assay (ELISA) technique. All biomarkers were measured and analyzed in the four age studies groups.

**Results:**

The expression of *TGF-βRI*, *TGF-βRII*, *IFNARI*, *IFNARII*, *IRF9*, and *SMAD3* was markedly upregulated in all age groups of patients compared with the matched control groups. Serum levels of IFN-α and SERPINE1 were significantly higher in patient groups than in control groups. While TGF-β serum levels were only significantly elevated in the 20 to 40 and over 60 years patient group than in matched control groups.

**Conclusions:**

These data showed that the age of patients, at least at the time of admission, may not significantly affect TGF-β- and IFN-I-associated immune responses. However, it is possible that the severity of the disease affects these pathway-mediated responses, and more studies with a larger sample size are needed to verify it.

**Supplementary Information:**

The online version contains supplementary material available at 10.1186/s12879-023-08225-9.

## Introduction

At the end of 2019, the acute respiratory syndrome of coronavirus 2 (SARS-CoV-2) became a threat to human health in the world, and the World Health Organization (WHO) declared the novel coronavirus disease 2019 (COVID-19) a pandemic [1]. After months, different variants of this virus are still causing the occurrence of new peaks [2]. The prognosis of patients with COVID-19 is very diverse, and about 50 to 80 percent of COVID-19 patients have only mild symptoms, such as a common cold, or are asymptomatic. In comparison, 20 to 50 percent of the patients develop severe respiratory and systemic syndromes that require immediate hospitalization and special care [3]. According to the latest studies, the death rate of COVID-19 varies from 1.7 to 13% in different parts of the world [3]. It is well-documented that the immune system and its components, such as immune mediators, play a critical role in the pathogenesis of SARS-CoV-2 infection [4, 5]. On the other hand, age could affect immune responses in patients with COVID-19 [6].

Cytokines and chemokines are involved in the pathogenesis of numerous human disorders [7–9]. Among these immune mediators, interferons (IFNs) are known as pleiotropic cytokines with antiviral properties and participate in immune regulation [10]. IFNs could have pro-inflammatory and anti-inflammatory roles in physiologic and pathologic conditions [11]. In patients with COVID-19, upon activating the IFN-type I (IFN-I)/ IFN-α/β receptor (IFNAR) pathway, IFN-α levels are increased, which ultimately may lead to viral clearance [12]. On the other hand, IFNγ, secreted from CD8^+^ cytotoxic T lymphocytes, can increase in line with innate antiviral immune responses in COVID-19 patients [13]. However, the production of IFNs faces inhibitory mechanisms by the virus and host immune system components. These factors facilitate infection progression and the inability of the host’s immune system to clear the virus effectively [12].

In most patients with COVID-19, death is occurred due to lung failure and acute respiratory distress syndrome (ARDS) [14]. This syndrome is characterized by cytokine storm, edema, and lung fibrosis in the late stages of COVID-19 [15]. Pulmonary fibrosis may be initiated mainly following the activation of the tumor growth factor-beta (TGF-β)/TGF-βR pathway and TGF-β-mediated SERPINE1 overexpression [16, 17]. SERPINE1 (plasminogen activator inhibitor-1[PAI-1]) is a member of the Serpin family and binds to tissue-type plasminogen (tPA) activator and urokinase plasminogen activator (uPA) [18]. It has been shown that human neutrophil elastase regulates the expression of SERPINE1 [19].

SERPINE1 inhibits plasmin forming and hinders fibrinolysis, as well as blood clot dissolution, resulting in balancing the fibrin degradation and plasminogen activation in physiologic conditions [19]. Emerging evidence revealed that normal fibrinolysis is suppressed in COVID-19-induced coagulopathy [20]. Previous studies on patients with COVID-19 demonstrated that reduced fibrinolysis was associated with SERPINE1 overexpression, and its elevation could lead to fibrin deposition in the lung parenchyma [21]. In addition, the upregulation of SERPINE1 is associated with severe lung disease and high mortality regardless of COVID-19 severity [22–24]. In addition, tPA levels were significantly elevated in patients with COVID-19. However, SERPINE1 overexpression can overcome elevated tPA levels [21]. According to the few studies available in the literature on the interaction of the IFN-I and TGF-β axes and the role of SERPINE1 in the induction of coagulopathy and pulmonary fibrosis, examining the expression of the involved molecules in these axes as well as their fluctuations, can open new windows to a better understanding of COVID-19 pathogenesis and emerging therapeutic approaches. Considering the role of IFN-I in activating the TGF-β pathway [25], as well as the effect of age on immune responses in the SARS-CoV-2 infection [26], this study investigated the expression of important molecules involved in IFN-I and TGF-β signaling pathways in different age groups. Moreover, the level of SERPINE1, which is the consequence of the TGF-β pathway activation, has been measured in different age groups of patients with COVID-19 compared with age-matched control groups [27].

## Methodology

### Subjects and study design

This age-matched case–control study involves 40 COVID-19 patients (cases) and 40 healthy controls. The male/female ratios were 20/21 for cases and 11/29 for controls. Enrolled subjects were categorized into four groups, including group 1 (up to 20 years, *n* = 10), group 2 (20–40 years, *n* = 10), group 3 (40–60 years, *n* = 10), and group 4 (over 60 years, *n* = 10) (Table 1). The present study was performed on patients admitted to Ali Ibn Abitaleb Hospital, Rafsanjan, Iran, from September 2021 until November 2021. Nasopharyngeal samples were collected from patients with clinical symptoms of SARS-CoV-2 infection for real-time (RT)-PCR assay. Only patients with verified molecular diagnoses were included in the study. Moreover, all subjects with other bacterial or viral infections, respiratory system-related illnesses, allergies, asthma, autoimmunity, malignancies, and immunocompromised patients were excluded from this study. A complete paraclinical examination and chest computed tomography (CT) scans were performed for all patients. Complete cell blood count (CBC), C-reactive protein (CRP) levels, body temperature, blood oxygen amount, prothrombin time test (PT), partial thromboplastin time (PTT), as well as biochemical indices, were assessed by appropriate methods and instruments. The demographic and clinical data of the enrolled patients are shown in Table 1.

**Table 1:** Control and patient demographic and clinical data

	**Control**				**Patients**			
	**C < 20**	**C 20–40**	**C 40–60**	**C > 60**	***P* < 20**	**P 20–40**	**P 40–60**	***P* > 60**
**Number of values**	9	10	10	11	11	11	8	11
**Minimum**	1	26	42	62	1	21	43	61
**Maximum**	18	39	56	80	17	40	60	92
**Range**	17	13	14	18	16	19	17	31
**Mean**	7.44	33.7	48.3	68.09	4.54	33.64	48.88	70.09
**Median**	5	35	46	67	2	36	47.5	67
**Std. Deviation**	6.98	4.24	6.05	5.7	5.1	6	5.38	9.77
**Sex (m/f)**	2/7	2/8	3/7	4/7	4/7	7/4	4/4	5/6
**Patient’s indices**	< 20 *n* = 11	20–40 *n* = 11	40–60 *n* = 8		> 60 *n* = 11		*P* value	
**spO**_**2**_**%**	0.90 ± 0.04	0.9 ± 0.02	0.88 ± 0.05		0.84 ± 0.05		0.001*	
***Median***	9	9	0.85		0.85			
**Severity *moderate/severe***	10/1	9/2	3/5		3/8		-	
**Body temperature *C***^***°***^	37.83 ± 0.66	38.31 ± 0.64	38.58 ± 0.62		38.17 ± 0.67		0.105	
***Median***	38	38.2	38.8		38.5			
**Respiratory rate *breaths per minute***	16.45 ± 4.08	17.45 ± 3.11	18.50 ± 5.09		20.45 ± 5.26		0.195	
***Median***	17	17	17.5		22			
**Heart rate (pulse) *bpm***	108.7 ± 13.91	99.27 ± 13.62	96.63 ± 13.88		100.4 ± 11.20		0.2	
***Median***	115	102	98		98			
**Systolic BP *mmHg***	12.27 ± 2.28	13 ± 1.09	12.25 ± 3.77		12.55 ± 2.11		0.8	
***Median***	12	13	12.5		13			
**Diastolic BP *mmHg***	7.5 ± 0.74	7.22 ± 1.14	7.6 ± 1.51		7.54 ± 1.45		0.9	
***Median***	7	7	8.25		8			
**HCO**_**3**_ ***mEq/L***	17.72 ± 1.46	22.98 ± 3.34	24.26 ± 5.92		20.95 ± 4.95		0.006 *	
***Median***	17	23	23		21			
**pCO**_**2**_ ***mmHg***	27.18 ± 3.18	34.32 ± 8.85	33.15 ± 9.33		29.33 ± 9.6		0.16	
***Median***	26	37.5	30		26			

*BP* Blood pressure, *C* Control, *P* Patients, *HCO*_*3*_ Bicarbonate, *pCO2*, Carbon dioxide

^*^Significant (*p* < 0.05

Based on the WHO-China Report for SARS-CoV-2 infection, patients were categorized into two categories according to the disease severity: moderate (*n* = 25) was distinct as more symptomatic with less than 50% radiological findings on chest CT scan, and SpO2 between 90 to 93%; severe disease (*n* = 16) was defined as respiratory distress with over 50% lung involvement and SpO2 less than 90% that may need mechanical ventilation or being admitted into the intensive care unit (ICU) [28]. Control blood samples were collected from healthy subjects without clinical symptoms or exposure to COVID-19 patients. The Rafsanjan University of Medical Sciences ethics committee approved the study protocol (IR.RUMS. REC.1400.149). Informed consent was obtained from all subjects. Furthermore, informed consent was obtained from parents or legal guardians for people under 16 years of age to include in the study.

### Gene expression assays

Five mL of peripheral blood was collected from all patients and healthy subjects in two fractions for RNA extraction and serum separation. Serum samples were stored at -20° C until further experiments. Total RNA was extracted from the whole blood specimens using the Karmania Pars Gene extraction Kit (Kerman, Iran). The integrity and purity of extracted total RNA were evaluated using gel agarose electrophoresis and spectrophotometric method. The 260/280 ratios were between 1.8 to 2 for all samples, showing a high purity and quality of the extracted RNAs and an optimal extraction procedure. Then, total RNAs were converted to complementary DNA (cDNA) using the One-Step RT-PCR Series Kit (KPG, Kerman, Iran) by 15µL ready-to-use cDNA master mix and a 5µL of 1 ng to 5 µg normalized RNA template. The protocol recommended by the manufacturer was: 42–50 °C for 30 min; 90 °C for 5 min; (reverse transcriptase (RT) enzyme inactivation), and lastly, the microtubes were chilled on the ice for 2 min.

The expression of target genes, including *TGFR1*, *TGFR2*, interferon regulatory factor 9 (*IRF9*), *IFNAR1*, *IFNAR2*, and SMAD family member 3 (*SMAD3*)*,* were quantified in whole blood cell samples isolated from patients with COVID-19 and matched healthy subjects using the 2X qPCRBIO SYGreen Mix Hi-ROX (PCRBiosystem, England). *Actin-β* was considered the reference gene. All reactions were performed in duplicate. Rotor-Gene Q 2plex System (Qiagen) according to the suggested protocol: 1 cycle of 95 °C for 2 min; 40 cycles of 95 °C for 5 s (denaturation), and 60–65 °C (annealing/Extension) for 20 to 30 s was used for templates amplification. Moreover, the melting curve step was considered for the final step by 10 s at 95 °C and then 10 s each at 0.2 °C enhancements between 62 and 95 °C. Table A in the supplementary material demonstrates the features of primers and amplified segments.

### Cytokine assays

To measure the serum levels of TGF-β (KPG, Kerman, Iran); SERPINE1 (R&D, #Cat No. DSE100, USA); and IFN-I (PBL Assay Science, #Cat No. 41100, USA) ELISA kits were used according to the manufacturer’s instructions. The ELISA kit’s assay range and sensitivity are presented in Table B (supplementary material). The data were only considered for further analysis when inter-and intra-assays values were CV < 15% and CV < 5%, respectively.

### Statistical analyses

GraphPad Prism 9 (GraphPad Software, San Diego, CA) was used for statistical analysis. The Shapiro–Wilk and one-sample Kolmogorov–Smirnov (KS) tests were employed to evaluate the variables’ normality. The studied group’s differences were also calculated using the independent sample T-test, Mann–Whitney U, and ANOVA (Tukey) tests. Moreover, the Pearson correlation test was used to estimate the association between age and serum levels of TGF-β, IFN-α, and SERPINE1 in patients with COVID-19. All data are presented as mean ± standard deviation (SD), and a *p*-value less than 0.05 was considered statistically significant. The 2^−ΔΔCt^ formula calculated the relative expression of the PCR products.

## Results

Demographic data and the number of patients and healthy people in each group are shown in Table 1. The results showed no significant difference between the mean age in different control groups and patients. Moreover, the analysis of laboratory data showed no significant difference between the number of white blood cells (WBCs) in different age groups of healthy and COVID-19 patients. In contrast, the number of lymphocytes in different age groups of patients showed a significant decrease compared to the matched groups of healthy people (*P* < 0.05). The mean serum level of CRP in all four age groups of patients showed a significant increase compared with the control groups (*P* < 0.05). In addition, the mean duration of PT and PTT tests in patients aged 20 to 40 years and over 60 years showed a significant increase compared to the age-matched control groups (*P* < 0.05). The results also showed that the mean serum levels of lactate dehydrogenase (LDH) in all four age groups of patients had a significant increase compared to the age-matched control groups (*P* < 0.05) (Table 2). The data analysis from the patients with COVID-19 showed a considerable difference in the amount of SpO_2_ (*P* = 0.001) and HCO_3_ (*P* = 0.006) in the four different age groups included in the study. However, in other indices, including body temperature, respiration rate (RR), pulse rate (PR), systolic and diastolic blood pressure, and pCO_2_, no significant difference was observed between different age groups of patients (Table 1).

**Table 2:** Demographic and clinical data of control and patient in different age groups

Groups	Subjects	Age year	WBC Per µL	Lymphocyte × 100	CRP mg/mL	PT s	PTT s	LDH IU/L
***Under 20 years***	**Control**	7.44 ± 6.98	5850.88 ± 1095.3	0.28 ± 0.04	4.57 ± 1.33	11.77 ± 0.83	25.44 ± 1.01	212.77 ± 34.82
	***n* = 9**							
	**Median**	5	6335	0.29	4	12	26	218
	**Patients**	4.54 ± 5.1	7218.18 ± 3982.66	0.17 ± 0.03	23.54 ± 1.36	13.12 ± 0.9	30 ± 7.5	604.54 ± 110.91
	***n* = 11**							
	**Median**	2	5000	0.17	22	13	30	620
***p* value**		0.29	0.33	< 0.0001*	< 0.0001*	0.002*	0.08	< 0.0001*
***20–40 years***	**Control**	33.7 ± 4.24	5960.1 ± 1437.4	0.25 ± 0.04	4.66 ± 1.64	11.6 ± 1.26	24.8 ± 1.22	203.13 ± 39.95
	***n* = 10**							
	**Median**	35	5514	0.27	4	12	25	206
	**Patients**	33.63 ± 6	10,354.54 ± 6561.3	0.16 ± 0.05	40.81 ± 20.32	13.32 ± 1.26	35.02 ± 12.27	692.27 ± 332.54
	***n* = 11**							
	**Median**	36	6600	0.16	37	13.1	35	678
***p* value**		0.97	0.052	< 0.0001*	< 0.0001*	0.005*	0.01*	< 0.0001*
***40–60 years***	**Control**	48.3 ± 6.05	5264 ± 1495.39	0.25 ± 0.03	6.19 ± 2.19	12 ± 1.33	25.1 ± 0.87	191.68 ± 46.97
	***n* = 10**							
	**Median**	46	4742	0.25	6	12	25	174
	**Patients**	48.87 ± 5.38	5187.5 ± 2699.96	0.19 ± 0.07	45.12 ± 10.66	13.32 ± 1.4	39.5 ± 9.08	888.12 ± 759.89
	***n* = 8**							
	**Median**	47.5	4250	0.21	48	13	38	635
***p* value**		0.83	0.93	0.002*	< 0.0001*	0.057	< 0.0001*	0.01*
***More than 60 years***	**Control**	68.09 ± 5.7	5124.36 ± 1293	0.28 ± 0.05	6.05 ± 2.34	11.36 ± 1.62	25.09 ± 0.94	183.83 ± 40.49
	***n* = 11**							
	**Median**	67	5148	0.29	6	11	25	178
	**Patients**	70.09 ± 9.77	7323.63 ± 4218.4	0.17 ± 0.06	37.81 ± 15.1	13.03 ± 1.41	36.5 ± 7.48	729.18 ± 173.72
	***n* = 11**							
	**Median**	67	6400	0.18	36	12.6	37	715
***p* value**		0.56	0.11	< 0.0001*	< 0.0001*	0.01*	< 0.0001*	< 0.0001*

*WBC* White blood cell, *CRP* C-reactive protein, *PT* Prothrombin time test, *PTT* Partial thromboplastin time, *LDH* Lactate dehydrogenase, *s* Second

^*^Significant (*p* < 0.05)

**Fig. 1:**
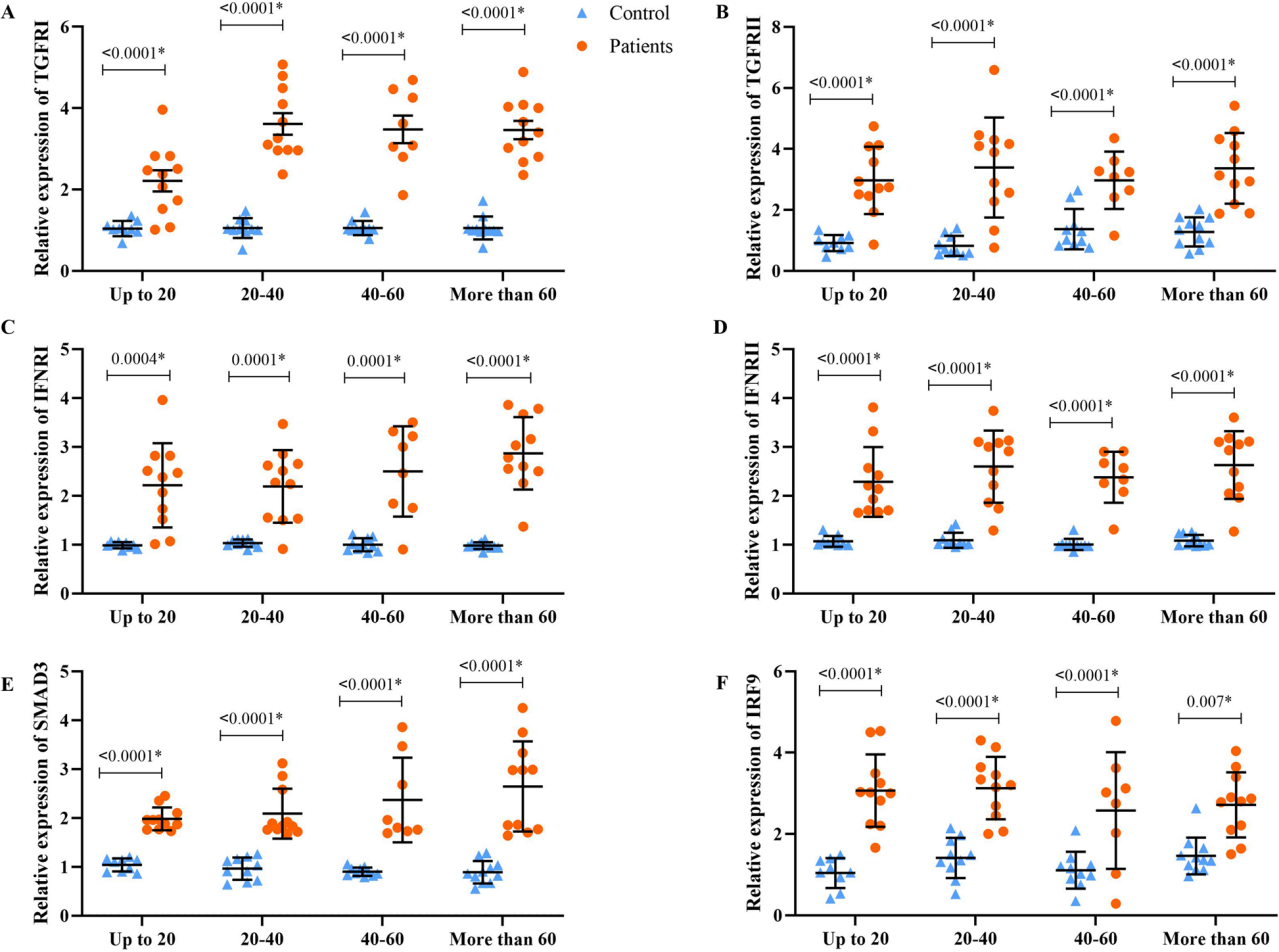
Demonstrates the alteration in the mRNA level of *TGF-βRI* (**A**), *TGF-βRII* (**B**), *IFNRI* (**C**), *IFNRII* (**D**), *SMAD3* (**E**), and *IRF9* (**F**) in different age groups of patients compared with matched control groups. The data are presented as mean ± SD. A *P* value less than 0.05 is considered statistically significant

The qPCR results showed that the expression of *TGF-βRI*, *TGF-βRII*, *IFNRI*, *IFNRII*, *IRF-9,* and *SMAD3* genes in all four groups of patients upregulated significantly compared to the control groups (*P* < 0.05) (Fig. 1).

**Fig. 2:**
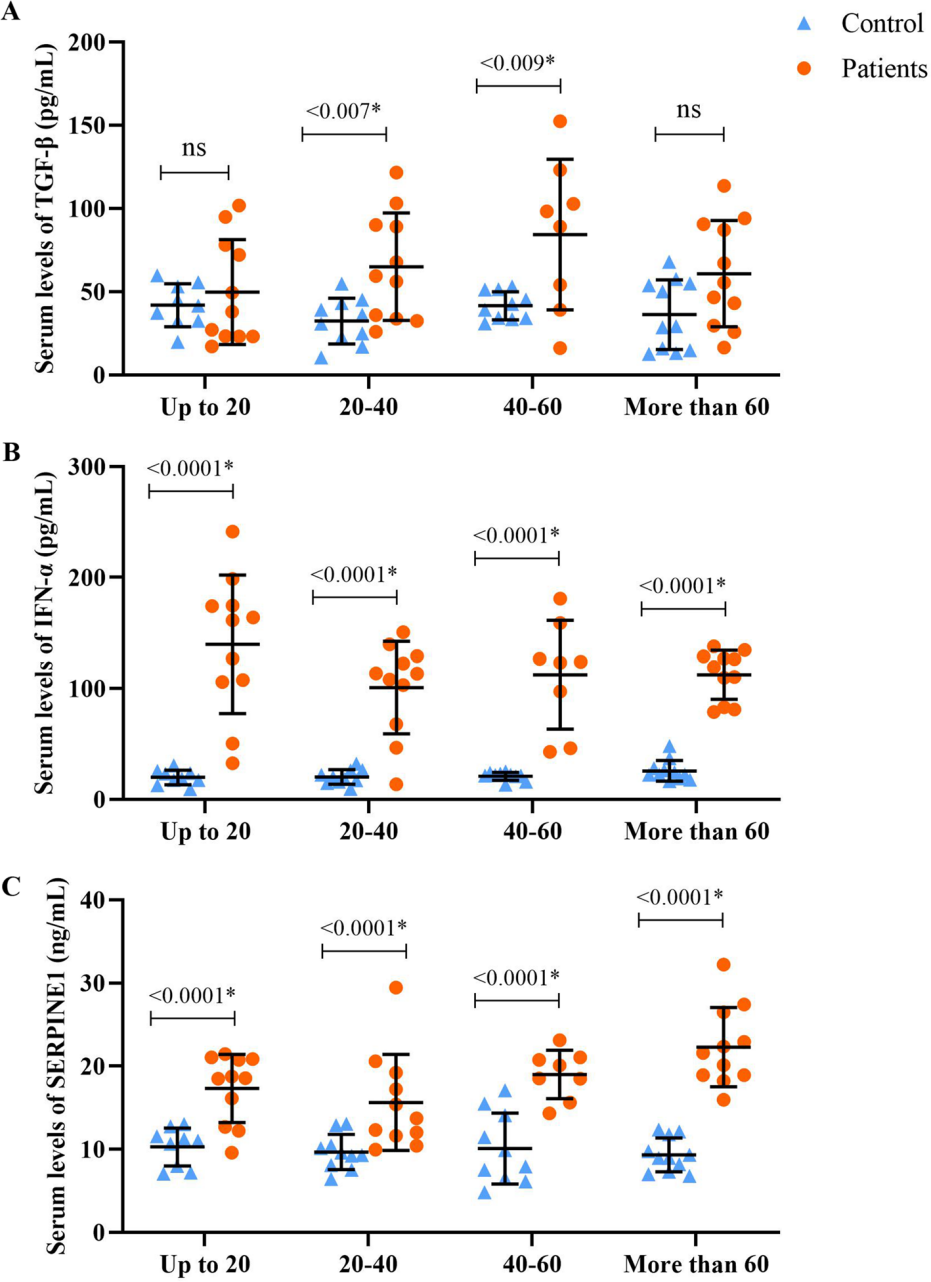
Demonstrates serum levels of TGF-β (**A**), IFN-α (**B**), and SERPINE1 (**C**) in different age groups of patients with COVID-19 compared with matched control groups. The data are presented as mean ± SD. A *P* value less than 0.05 is considered statistically significant

**Fig. 3:**
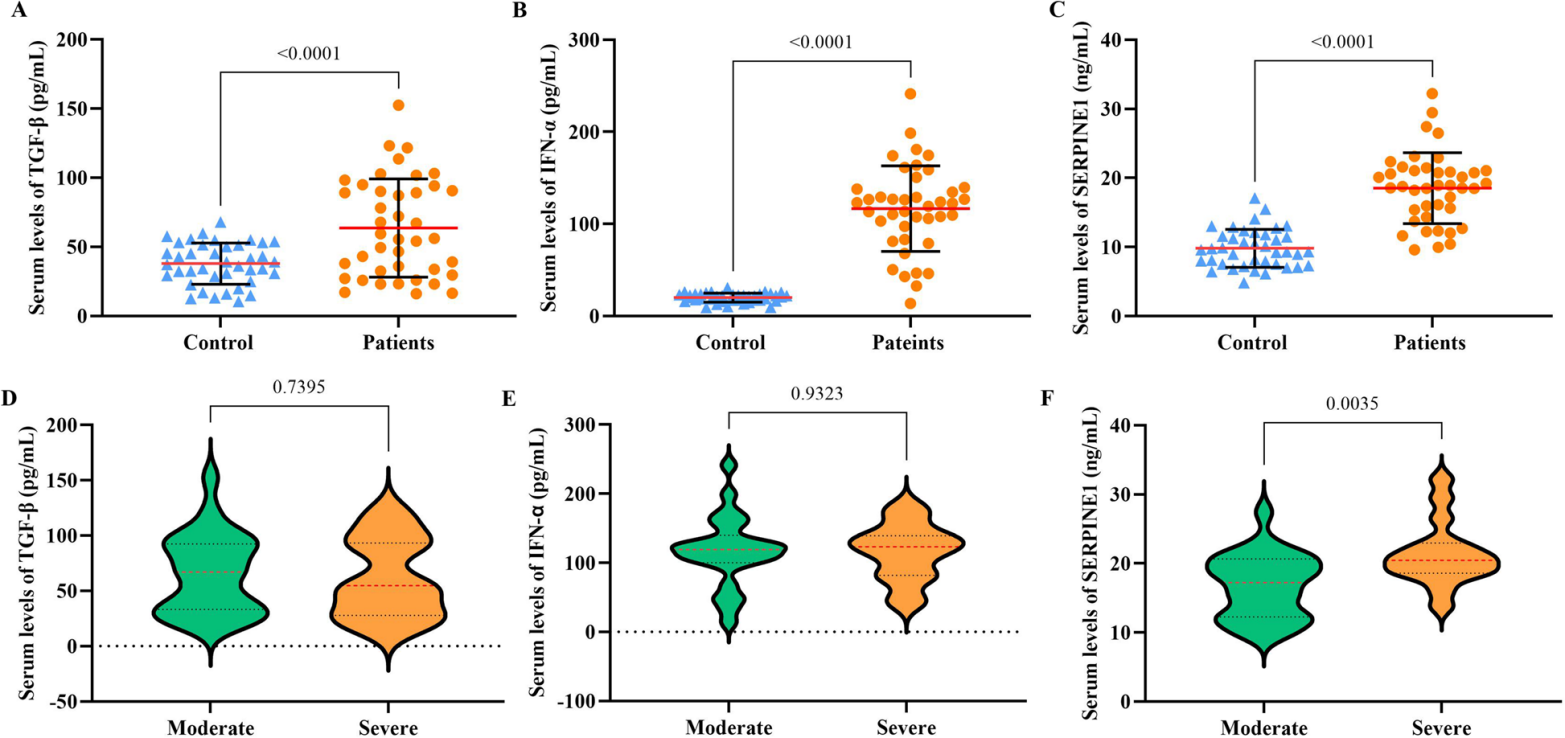
Illustrates serum levels of TGF-β (**A**), IFN-α (**B**), and SERPINE1 (**C**) in all enrolled patients compared with all healthy subjects in the control group. In addition, serum levels of TGF-β (**D**), IFN-α (**E**), and SERPINE1 (**F**) are shown based on disease severity (moderate/severe). The data are presented as mean ± SD. A *P* value less than 0.05 is considered statistically significant

**Fig. 4:**
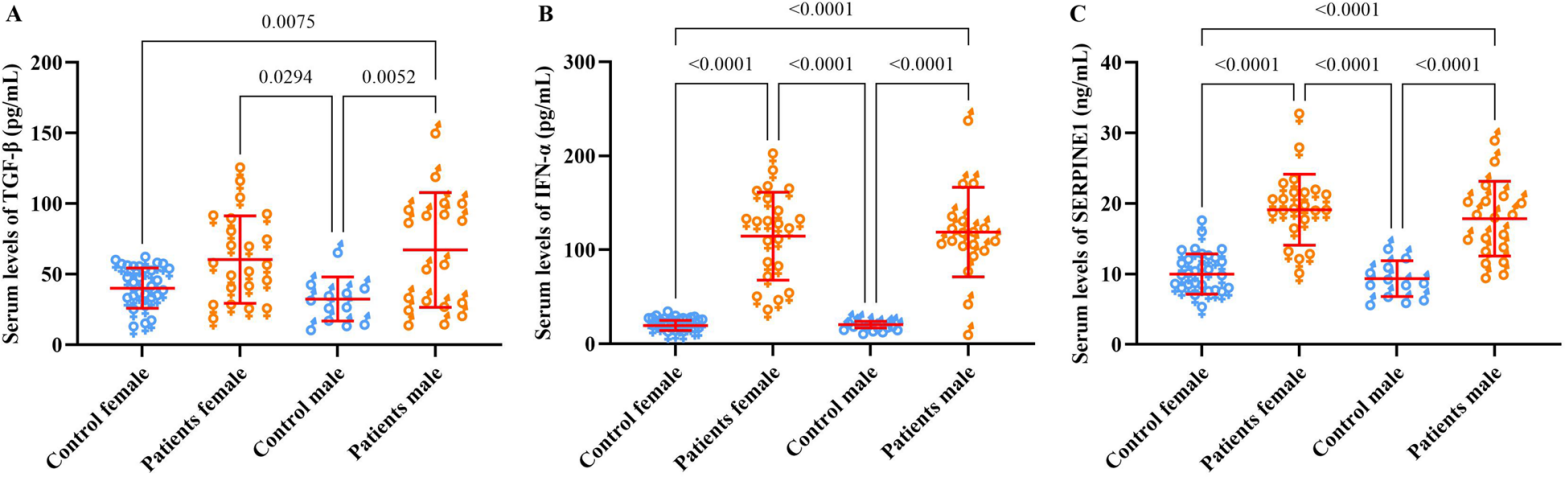
Illustrates serum levels of TGF-β (**A**), IFN-α (**B**), and SERPINE1 (**C**) according to the gender (female/male) of the patient and control groups. The data are presented as mean ± SD. A *P* value less than 0.05 is considered statistically significant

The analysis of TGF-β, IFN-α, and SERPINE1 in different patients and healthy individuals also revealed that the mean serum level of TGF-β was only elevated in the 20–40 and 40–60 years patients than in matched control groups (Fig. 2A). Regardless of age, the difference in the mean serum levels of TGF-β was significantly higher in patients than in control (*P* < 0.0001) (Fig. 3A). In addition, there was a significant difference in serum levels of IFN-α and SERPINE1 both in different age groups, regardless of age between the patient and control groups (*P* < 0.0001) (Fig. 2B, C and 3B, C). The severity of the disease did not affect the serum level of TGF-β and IFN-α (Fig. 3D, E), while the mean serum level of SERPINE1 was higher in patients with severe disease than in the moderate group (*P* = 0.0035) (Fig. 3F). The mean serum levels of TGF-β, IFN-α, and SERPINE1 were not significantly different between male and female patients (Fig. 4).

**Fig. 5:**
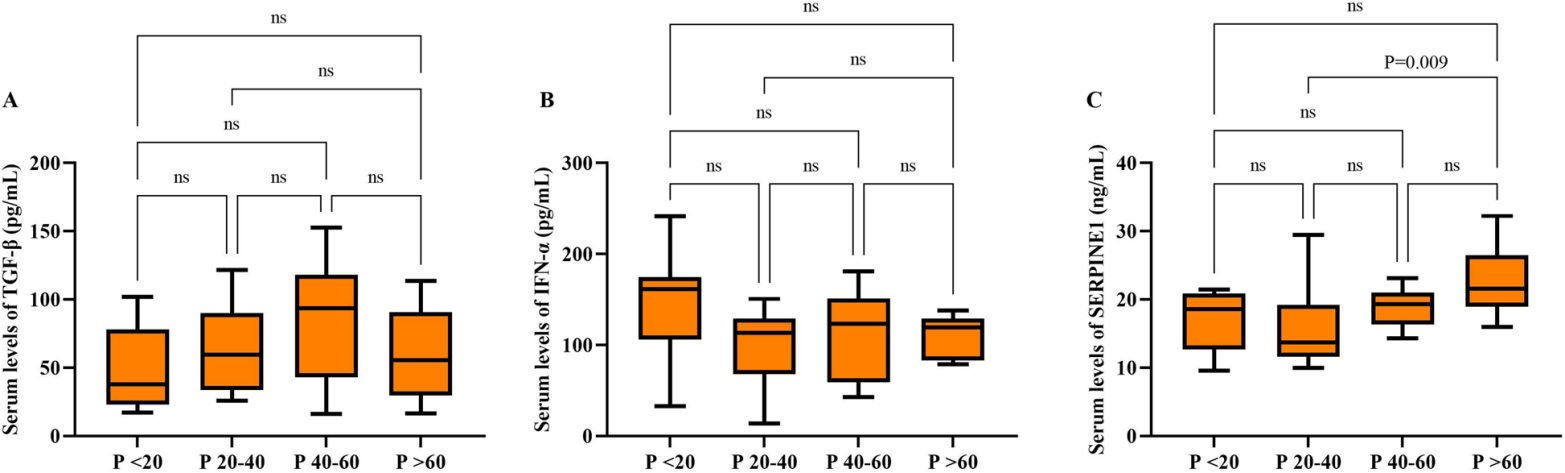
Multiple comparisons between the mean serum levels of TGF-β (**A**), IFN-α (**B**), and SERPINE1 (**C**) in four age groups of patients (*P* < 20; patients under 20 years, P20-40; patients between 20 to 40 years, P40-60; patients between 40 to 60 years, *P* > 60; patients above 60 years). The data are presented as mean ± SD. A *P* value less than 0.05 is considered statistically significant. P; patient, ns; non-significant

**Fig. 6:**
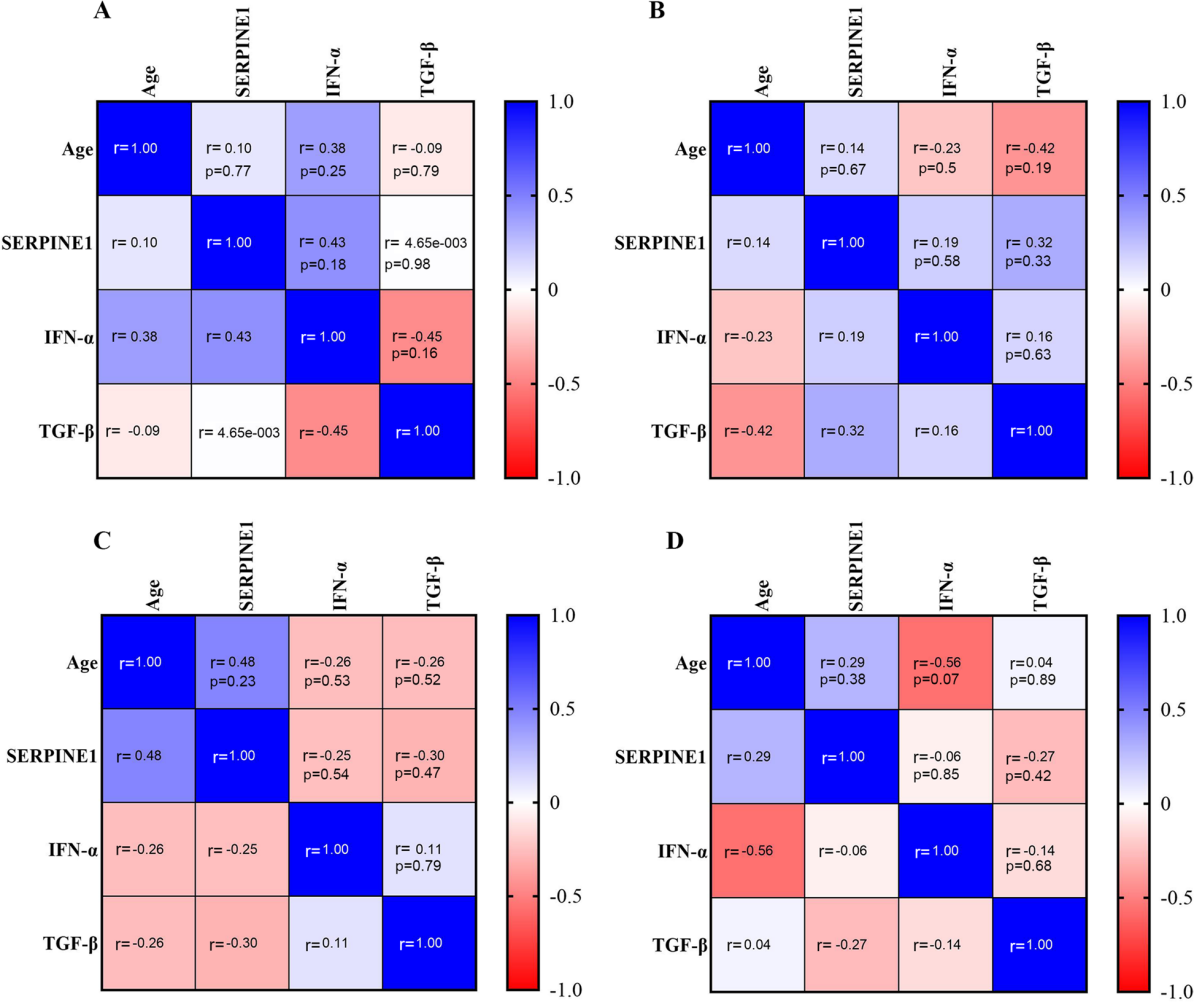
The correlations between the age and TGF-β, IFN-α, and SERPINE1 serum levels in four age groups of patients **A** (patients under 20 years), **B** (patients between 20 to 40 years), **C** (patients between 40 to 60 years), **D** (patients above 60 years). The Pearson correlation coefficient (r) and p values are shown in each cell, and the range of the confidence interval of r (between -1 and 1) is illustrated in the side box of the graphs. There was no significant correlation between the variables in different age groups. A *p*-value less than 0.05 is considered statistically significant

Tukey’s test was used for multiple compression between the mean serum levels of TGF-β, IFN-α, and SERPINE1 in four studied age groups of patients, and significant differences are shown in Fig. 5. Furthermore, the data analysis related to the serum levels of TGF-β, IFN-α, and SERPINE1 in different age groups showed no significant correlation between these markers and the age of the patients (Fig. 6).

## Discussion

In this study, the effect of SARS-CoV-2 infection on IFN-α and TGF-β cytokine axes and the interaction of these axes were investigated, focusing on age. Type-I interferons (IFN-Is), including IFN-Iα and IFN-Iβ, are at the forefront of host anti-viral innate immune responses [29]. IFN-Is can exert their antiviral effects on virus-infected bronchial epithelial cells by inducing the expression of IRF9 and interferon-stimulated genes (ISGs) in an endocrine and paracrine fashion [12]. Although IFN-Is often cannot be effective in virus clearance because of various reasons such as virus escape mechanisms, anti-interferon antibodies, and interaction with multiple cytokines [12, 30]. The results of the present study showed that the gene expression of *IFNRI*, *IFNRII, and IRF9* was significantly upregulated in the peripheral blood cells of patients with COVID-19 in all four age groups under 20 years, 20 to 40 years, 40 to 60 years, and over 60 years compared to aged-matched control groups. Furthermore, the serum level of INF-α was increased significantly in all four age groups of patients compared to the control groups, which shows that its level is high at the time of admission. Additionally, in patients with severe disease, the level of IFN-α was not higher than in patients with moderate disease severity, and the gender of the patients did not affect the levels of this cytokine. However, the sample size may affect these results.

It has been reported that immunosuppressive cytokines, such as TGF-β and IL-10, increased following SARS-CoV-2 infection [31, 32]. TGF-β, a multifunctional cytokine, can regulate various biological mechanisms, including cell apoptosis, proliferation, differentiation, tissue repair, fibrosis, and immune responses [33]. Following viral infections, alteration in the TGF-β pathway inhibits cell apoptosis and induces fibroblast proliferation and myofibroblast differentiation, developing pulmonary fibrosis [34]. Lung biopsies obtained from patients with SARS-CoV-2 infection showed moderate inflammation and mild fibrosis [35]. Moreover, in the early stage of SARS-CoV-2 infection, robust inflammatory responses and dysregulation of the fibrinolytic and coagulation pathways can promote the activation of the latent form of TGF-β in the blood circulation and lungs [36]. These data suggest that overexpression of TGF-β in patients with COVID-19 might lead to pulmonary fibrosis and lung dysfunction [37].

On the other hand, TGF-β induces the expression of SERPINE1, accumulating fibrin, stimulating coagulation, clot formation, and thrombosis [38]. The findings of this study demonstrated that the gene expression of *TGF-βRI*, *TGF-βRII*, and *SMAD3* was increased in patient groups compared with control groups. Furthermore, serum levels of TGF-β in patients with COVID-19 increased significantly compared to the control group. However, this increase was not associated with the severity of the disease and the gender of the patients. In different age groups, the serum level of TGF-β in the age groups of 20 to 40 years and 40 to 60 years had a significant difference compared to the control groups in the same age ranges. In people under 20 years old and over 60, the levels of TGF-β were not significantly different compared to the control group.

Furthermore, SERPINE1 levels in all four age groups of patients increased significantly compared to the control groups. However, unlike TGF-β, this increase depended on the disease severity, and peripheral SERPINE1 levels were substantially higher in patients with severe disease than in patients with moderate disease. In parallel with these findings, other studies on patients with SARS-CoV-2 infection reported that serum levels of SERPINE1 were increased markedly than healthy subjects, which is another hint that normal fibrinolysis is impaired in COVID-19-induced coagulopathy [20, 21, 39]. It has also been demonstrated that SAMD3 can induce the expression of SERPINE1 [40].

Interestingly, emerging evidence revealed that the N protein in SARS-CoV-2 could induce the expression of SMAD3 directly and independently of the TGF-β axis [41]. Therefore, in this way, SARS-CoV-2 infection can upregulate SERPINE1, inducing coagulopathy and pulmonary fibrosis. However, despite the increase in PT and PTT time in patients with COVID-19, our findings showed no significant correlation between the levels of SERPINE1 and PT or PTT duration [42]. Nevertheless, these results are obtained from COVID-19 patients at admission. They should be measured on other days or after discharge with larger sample sizes because pulmonary fibrosis is a long-term process and occurs in the post-COVID-19 era [43]. Increased TGF-β and SERPINE1 serum levels at admission may indicate the foundation for the initiation of fibrotic processes as well as COVID-19-mediated coagulopathies.

On the other hand, the question is whether the increase of IFN-Is in viral infections always favors the patient’s recovery. Recently, it has been revealed that there is an interaction between the IFN-I and TGF-β pathways, and increasing IFN-Is can induce the TGF-β signaling pathway [25]. As a result, increased IFN-α in SARS-CoV-2 infection may induce the TGF-β pathway and further pulmonary fibrosis-mediated mechanisms.

Our finding showed no significant difference between TGF-β and IFN-α serum levels in different age groups of patients with COVID-19. In patients over 60 years, the mean SERPINE1 level was significantly higher than the 20–40 years group. Nonetheless, there was no significant difference in the mean SERPINE1 level between other age groups. These findings indicate that despite the difference between different age groups in COVID-19 patients, the age, at least at the time of admission, may not significantly affect the serum levels of TGF-β, IFN-α, and SERPINE1. However, hospitalization time and sample size may affect and change these outcomes.

### Strengths and limitations

One of the strong points of this study design is focusing on different age groups of patients with COVID-19 at admission and before starting any treatment. Due to the specific conditions of the patients as well as different discharge times, it was almost impossible to obtain blood samples on other days of hospitalization. Routine treatments for SARS-CoV-2 infection also could affect the findings. Additionally, the small sample size was one of our challenges in this investigation because, in Rafsanjan city, where the study was conducted, the rate of COVID-19 infection and hospitalizations varied at different times. Besides, enrolling patients with COVID-19 with a broad age range and matching them with healthy subjects, especially people under 20 and over 80 years old, was challenging.

## Conclusion

In this study, we explored the impact of age on immune responses in SARS-CoV-2 infection, particularly on the interaction between the TGF-β and IFN-I axes. The findings of this study showed that the age of patients, at least at the admission time, may not significantly affect TGF-β and IFN-I-associated immune responses. However, the severity of the disease may affect these pathways and related immune responses. It appears that further studies with larger sample sizes at different times can effectively clarify the role of TGF-β and IFN-I axes in antiviral responses as well as SERPINE1-mediated coagulopathy and pulmonary fibrosis in patients with COVID-19.

## Supplementary Information

**Additional file 1:** **Table A.** The sequences of primers used in the study. **Table B.** Utilized ELISA kits specifications.

## Data Availability

The datasets used and/or analyzed during the current study are available from the corresponding author upon reasonable request.
