# Supplementary material for: Evaluation of the interaction between tumor growth factor-β and interferon type I pathways in patients with COVID-19: focusing on ages 1 to 90 years
Source: BMC Infect Dis. 2023 Apr 18;23:248. doi: 10.1186/s12879-023-08225-9 (PMC10112317; doi:10.1186/s12879-023-08225-9)
Supplement: Supplementary file 1 — Additional file 1: Table A. The sequences of primers used in the study. Table B. Utilized ELISA kits specifications. [file 12879_2023_8225_MOESM1_ESM.docx]

**SUPPLEMENTARY MATERIAL**

Table A. The sequences of primers used in the study.

| **Gene** | **Sequence (5'->3')** | **Annealing temperature (C°)** | **Product length (bp)** |
| --- | --- | --- | --- |
| ***TGFRI*** | ACTCAGTCAACAGGAAGGCA  CACTGTTGCCAAAGGAAGCT | 60 | 155 |
| ***TGFRII*** | AAGGAAGGGACCCATGACAG  ATGGCCAGAAGAGAAGTGCT | 60 | 179 |
| ***IFNRI*** | CGCTCTTAAGACATGGCTGG  TGTGAGACTGAAGAGCCTCC | 60 | 152 |
| ***IFNRII*** | TGCAAGTGTTCTCCAAGGGA  GCTTCCTTCAAAGTGCACCA | 60 | 146 |
| ***IRF9*** | TAAGGAGGGGGACACAGGAG  ACCTTGTAGGGCTCAGCAAC | 60 | 126 |
| ***SMAD3*** | AGAACCAAACCTCAACACAGC  TTCTGCTTTCCCTACCCACTT | 60 | 151 |
| ***Actin B*** | AAACTGGAACGGTGAAGGTG  AGAAGTGGGGTGGCTTTTAG | 60 | 169 |

Table B. Utilized ELISA kits specifications

| Marker | Brand | Cat No. | Assay range | Sensitivity |
| --- | --- | --- | --- | --- |
| TGF-β | KPG | KPG-HTGF | 10-200 pg/mL | 6 pg/mL |
| SERPINE1 | R&D System | DTSE100 | 0.3 - 20 ng/mL | 0.046 ng/mL |
| IFN-α | R&D System | 41100-1 | 156.0 - 5000 pg/mL | 12.5 pg/mL |
